# Combined extracorporeal shock wave lithotripsy and endoscopic treatment for pain in chronic pancreatitis (SCHOKE trial): study protocol for a randomized, sham-controlled trial

**DOI:** 10.1186/s13063-020-04296-0

**Published:** 2020-04-16

**Authors:** Søren S. Olesen, Asbjørn M. Drewes, Rajesh Gaud, Manu Tandan, Sundeep Lakhtakia, Mohan Ramchandani, G. V. Rao, D. Nageshwar Reddy, Rupjyoti Talukdar

**Affiliations:** 1 Department of Gastroenterology and Hepatology, Centre for Pancreatic Diseases, Aalborg University Hospital, Aalborg, Denmark; 2 Department of Medical Gastroenterology, Asian Institute of Gastroenterology, Hyderabad, Telangana India; 3 Department of Surgical Gastroenterology, Asian Institute of Gastroenterology, Hyderabad, Telangana India; 4 Pancreas Research Group, Wellcome DBT Labs, Asian Healthcare Foundation, Hyderabad, Telangana India

## Abstract

**Background:**

Pain is the primary symptom of chronic pancreatitis (CP) and remains a considerable therapeutic challenge. In patients with obstruction of the pancreatic duct, including stones and strictures, endoscopic treatment with or without preceding extracorporeal shock wave lithotripsy (ESWL) has been used for pancreatic duct decompression. The rationale for these procedures is based on the assumption that obstruction of the pancreatic duct leads to ductal hypertension and pain. However, clinical pain symptoms correlate poorly with pancreatic duct morphology, and the evidence for pancreatic duct decompression as an effective treatment for pain is based on case series and comparison between different procedures. No randomized, prospective, sham-controlled trials are currently available. The SCHOKE (Extracorporeal Shock Wave Lithotripsy and Endotherapy for Pain in Chronic Pancreatitis) trial is a randomized, sham-controlled trial designed to determine if pancreatic duct decompression is an effective treatment for pain in patients with CP.

**Methods:**

The SCHOKE trial is a randomized, single-blind, parallel-group, sham-controlled trial designed to evaluate the effect of combined ESWL and endoscopic treatment for pain in patients with CP. In total, 106 adult patients with painful CP and pancreatic duct obstruction will be randomized to combined ESWL and subsequent endoscopic treatment or corresponding sham procedures. The primary outcome is pain relief during the 3-month postrandomization period as documented in a pain diary. Secondary outcomes include quality of life and functional scores, patient global impression of change, change in use of analgesics, frequency of hospitalization, and complications. Standard follow-up is at 3 and 6 months after randomization. In an experimental substudy, quantitative sensory testing obtained before and after intervention will be used to obtain information on central pain processing and to develop models for prediction of treatment outcome.

**Discussion:**

The SCHOKE trial investigates if pancreatic duct decompression, obtained by combined ESWL and endoscopic treatment, is effective for pain treatment in patients with CP.

**Trial registration:**

ClinicalTrials.gov, NCT03966781. Registered on May 25, 2019.

**Protocol date and version identifier:** March 1, 2020; version 3.0.

**Sponsor:** Rupjyoti Talukdar, Department of Medical Gastroenterology, Asian Institute of Gastroenterology, Hyderabad, Telangana, India.

## Introduction

Pain is the primary symptom of chronic pancreatitis (CP) and remains a considerable therapeutic challenge [1]. In patients with pathological changes of the pancreatic duct, including stones and strictures, endoscopic treatment with or without preceding extracorporeal shock wave lithotripsy (ESWL) and surgery has been used with varying success to treat pain [2–4]. The rationale for such invasive procedures is based on the hypothesis that obstruction of the pancreatic duct leads to ductal hypertension and pain [5, 6]. However, clinical pain symptoms correlate poorly with pancreatic ductal morphology, as assessed by cross-sectional imaging [7, 8], and the response to endoscopic or surgical treatment is unpredictable, with long-term response rates ranging from 30% to 60% [3, 9, 10]. Also, the evidence for these treatments is based on case series and comparison between different procedures; no randomized, prospective, sham-controlled trials have evaluated the effectiveness of invasive treatments for pain in CP [9–11]. Additionally, a marked placebo effect has been observed in most trials of painful CP, and this, together with the natural history of disease, needs consideration when treatment effects are evaluated [12–14]. Therefore, the evidence for invasive treatments for pain in CP can be questioned [14].

Recent meta-analyses have documented that nonspecific effects of invasive procedures are generally large, particularly in the field of pain-related conditions and for endoscopy-based therapies [15, 16]. For example, endoscopic retrograde cholangiopancreatography (ERCP) with sphincterotomy (biliary or pancreatic sphincterotomy or both) has for many years been considered the state-of-the-art treatment for patients with abdominal pain due to suspected sphincter of Oddi dysfunction. However, a high-quality randomized controlled trial (RCT) showed that patients who had no sphincterotomy at ERCP, and who were blinded to their treatment, reported as much pain relief as those who underwent sphincterotomy [17, 18]. These findings challenge conventional wisdom and underscore the necessity for appropriately conducted RCTs to include a sham procedure when the effectiveness of invasive procedures is evaluated [14].

Albeit endoscopic treatment or surgery is widely used for pain in CP, these treatments are only effective in a subset of patients [4, 9, 19, 20]. An improved understanding of the mechanisms underlying pain in CP suggests that the pain etiology in most patients is multifactorial, and, in addition to the proposed mechanisms for pain (ductal obstruction/hypertension), a large body of evidence supports a “neuropathic pain phenotype” with abnormal processing in the peripheral and central neural pathways [6, 21, 22]. Invasive procedures will not be effective in neuropathic pain and can even be considered harmful [14]. This likely explains the variable response to endoscopic and surgical treatments and underlines an unmet need for biomarkers to identify responders to the different treatment modalities.

Quantitative sensory testing (QST) can be used to investigate the state of the pain system. The technique is based on the rationale that different neural pathways and networks can be explored using standardized stimulation with simultaneous recording of the evoked pain response by psychophysical and/or objective methods [23]. Due to spinal convergence between visceral afferents from the pancreas and somatic afferents from the T10 skin dermatome, somatic QST can reliably be used to assess if the pain system is locally sensitized by nociceptive input from the pancreas (segmental sensitization) [24, 25]. However, in many patients with chronic pain, the pain system has become dysfunctional and has undergone widespread sensitization, which is evident as abnormal responses (hyperalgesia) to stimuli applied in areas remote from the pancreas [26]. Taken together, QST profiling based on testing in several dermatomes, together with specific test paradigms (temporal summation and assessment of descending inhibition), can be used to determine whether patients have abnormal central pain processing or evidence of segmental or widespread sensitization [27–29].

## Methods

The study protocol is reported in accordance with the Standard Protocol Items: Recommendations for Interventional Trials (SPIRIT) guidelines (Additional file 1).

### Study hypothesis and aim

The hypothesis of the study is that pancreatic duct decompression following ESWL and endoscopic treatment induces short-term (3 months) and midterm (6 months) pain relief in patients with CP compared with a sham procedure. In addition, we hypothesize that QST can be used to predict the outcome of treatment. Hence, patients with evidence of widespread sensitization of central pain pathways are hypothesized to have a worse outcome after ESWL and endoscopic treatment than patients with no evidence of widespread sensitization.

### Study design and setting

We are conducting a randomized, single-blind, single-center, parallel-group, sham-controlled, prospective trial designed to evaluate the effect of combined ESWL and endoscopic treatment for pain in adult patients with CP. The study will be conducted at the tertiary care academic Asian Institute of Gastroenterology in Gachibowli, Hyderabad, India.

### Inclusion criteria

- A diagnosis of chronic calcific pancreatitis diagnosed using the Mayo Clinic diagnostic criteria [30]. Both diabetic and nondiabetic patients will be allowed to enter the study.
- Age ≥ 18 years.
- Chronic abdominal pain characteristic for CP with a pain intensity > 3 on a 0–10 visual analogue scale (VAS) and meeting the criteria for chronic pain (pain ≥ 3 days per week for ≥ 3 months).
- Obstruction of the pancreatic duct due to intraductal stones with dilation of the duct proximal to the obstruction, as determined by magnetic resonance cholangiopancreatography (MRCP) or abdominal computed tomography (CT).
- The patients must be able to read and understand the provided informed consent form.
- Patients must personally sign and date the informed consent document, indicating that he/she has been informed of all pertinent aspects of the trial.
- Patients should be willing to comply with the scheduled visits, clinical and experimental assessment plans, and other trial procedures.

### Exclusion criteria

- Patients with any clinically significant laboratory abnormalities that, in the opinion of the investigator, may increase the risk associated with trial participation or may interfere with the interpretation of the trial results.
- Previous history of pancreatic surgery, ESWL, or ERCP.
- Patients with a pancreatic stricture on cross-sectional imaging prior to study enrollment.
- Active alcohol or illicit drug dependency.
- Patients with evidence or history of medical or surgical disease of importance for this study as judged by the investigator.
- Patients must not have painful conditions other than CP that make them unable to distinguish the pain associated with CP from chronic pain of other origin.
- Presence of pancreatic head mass, multiple strictures, large ascites, and/or large fluid collections.

### Interventions

Patients will be randomly allocated to either combined ESWL and endoscopic treatment or sham treatment.

#### Combined ESWL and endoscopic treatment

Patients enrolled in the active treatment group will be subjected to ESWL followed by ERCP. In India, patients with CP typically present with large pancreatic stones that tend to be dense and spiculated. In order to ensure complete stone clearance, ESWL and ERCP are therefore performed together and in agreement with clinical practice in most centers [10, 31].

ESWL will be conducted with the patient under epidural anesthesia. For epidural anesthesia, bupivacaine will be used to block the T6–T12 thoracic spinal segments. The patient’s eyes will be lightly covered during the procedure. Once epidural anesthesia is achieved, the patient will be given a light sedation, and ESWL will be performed using a third-generation Dornier dual focus lithotripsy system (Dornier Delta 3; Dornier MedTech GmbH, Weßling, Germany), providing a maximum of 5000 shocks at the rate of 90 shocks per minute. If complete stone clearance is not achieved during the first ESWL session, a second session will be scheduled the following day.

After lithotripsy, stone fragments will be removed during an ERCP procedure. An endoscopic pancreatic sphincterotomy will be performed, and complete stone removal will be attempted with registration of pancreatic duct clearance. If cannulation fails after a maximum of five attempts, the patient will be subjected to precut sphincterotomy. In case a pancreatic duct stricture is identified during the ERCP procedure that was not detected by MRCP prior to enrollment, the stricture is dilated and followed by pancreatic stent insertion. A pancreatic duct stent will also be inserted in case of incomplete stone removal during the ERCP procedure.

Patients undergoing pancreatic duct stenting will be referred for a new ERCP procedure after 6 months for stent exchange or removal (after completion of all study assessments). When complete runoff of contrast material is observed after removal of the stent and an extraction balloon can be passed through the pancreatic duct, endoscopic treatment is considered completed, and further stenting will be stopped. Persistent strictures will be treated by repeated endoscopic dilations and sequential insertion of new stents in agreement with the European Society of Gastrointestinal Endoscopy guidelines [32].

#### Sham treatment

In the sham/control group, patients will be given a transient superficial pinprick sensation to give the feeling of epidural anesthesia prior to sham ESWL. Subsequently, the lithotripsy machine will be switched on, without establishing any form of contact with the patient’s body. The patient’s eyes will be lightly covered during the entire procedure. Following sham ESWL, patients will be subjected to sham ERCP with duodenal intubation of the endoscope and examination of the papillary area, but no pancreatic ductal intervention will be performed.

#### Concomitant medication

Patients will be instructed not to change their regular pain treatment during the trial period. Regular pain treatment will be recorded twice: at the screening visit and at the last visit. Rescue pain medication, taken on an “as needed basis,” is allowed throughout the trial period, and its use will be documented and quantified in the pain diary. Patients with exocrine pancreatic insufficiency will remain on enzyme replacement therapy during the trial period and follow-up.

### Outcomes

The study consists of a clinical part and an experimental part. The clinical part of the study aims to investigate the pain-relieving effects of combined ESWL and endoscopic treatment in patients with painful CP in comparison with sham treatment. The experimental part of the study aims to evaluate if QST profiles obtained prior to ESWL and endoscopic intervention can be used for prediction of treatment outcome.

#### Primary clinical endpoint

The primary clinical endpoint is pain relief. Average and maximal daily clinical pain intensity scores will be recorded in a patient pain diary based on a 0–10 VAS, with registration of the baseline pain intensity scores done in the week prior to intervention and weekly recordings continued for a 3-month period after intervention. Mean values of pain scores will be calculated over 1 week to adjust for day-to-day variability in pain intensity. The difference in pain scores between patients receiving active treatment (ESWL and ERCP) and sham treatment are compared, with the primary comparison of average pain scores 3 months after intervention. Weekly telephone interviews from a study coordinator will be undertaken to facilitate accurate registration and compliance pain score.

#### Secondary clinical endpoints

- Difference between groups in pain scores after 6 months.
- The ratio of responders versus nonresponders defined by a decrease in the average clinical pain score (VAS) of 30% after 3 and 6 months compared with baseline.
- Difference between groups in number of pain-free days after 3 and 6 months.
- Change in analgesic consumption (if used) after 3 and 6 months compared with baseline.
- Difference between groups in total number of hospitalizations during the study period.
- Difference between groups in total duration of hospital stays during the study period.
- Difference in total loss of working days due to CP between groups during the study period.
- Difference between groups in cumulative cost attributed to CP-related treatment and disability (loss of working days) during the study period.
- Difference between groups in quality of life using the European Organization for Research and Treatment of Cancer Quality of Life questionnaire after 3 and 6 months [15].
- Difference between groups in pain and physical functioning composite scores of the modified Brief Pain Inventory–short form after 3 and 6 months [16].
- Difference between groups in depression and anxiety scores of the Hospital Anxiety and Depression Scale after 3 and 6 months [33].
- Patient global impression of change after 3 and 6 months [18].
- Assessment of complications resulting from interventions during the study period.

#### Experimental endpoints

The following experimental pain measures will be employed prior to intervention as well as 24–48 h and 3 months after intervention to characterize changes in pain processing induced by the assigned procedures:

- Muscle pressure stimulation (pancreatic viscerotome [T10 ventral and dorsal] and control areas [C5, L1 and L4])
- Bone pressure stimulation (tibia bone)
- Temporal summation to repetitive pinprick stimulations of the pancreatic area (T10) and control area [dominant forearm])
- Conditioned pain modulation (CPM)

### Sample size determination

The study is powered to detect a minimal difference between groups of 30% in the average clinical pain score 3 months after intervention [34]. On the basis of an assumed SD of 45%, we determine that a study with 48 patients per group is needed to provide power of 90% (to allow secondary endpoints) with the use of a two-sided significance level of 0.05 [35]. To allow a 10% dropout rate, the sample size is set at 106 patients.

### Randomization, blinding, and treatment allocation

Randomization is performed using an automatic assignment system that conceals allocation. Block randomization is employed with randomization of six patients per block to equal proportions for sham procedures or combined ESWL and endotherapy. No stratification of randomization based on demographic or clinical variables will be applied. The allocation sequence will be generated by a biostatistician who will not be involved in the administration of treatment or recording of data. Randomization of patients into the two groups will be done by a physician who will not have access to the clinical data of the patients.

The patients will be blinded to the treatment received. The patients in the sham procedure will be provided a superficial needle prick with a sterile needle to mimic epidural anesthesia. Then the ESWL instrument will be started so that the patients get an auditory perception that the treatment is being provided. The patient’s eyes will be covered, and if the patient wishes, he or she will be given light sedation. The operator cannot be blinded, owing to the nature of the study. However, the principal investigator and the statistician will be blinded to the treatment arms.

In case of an emergency that necessitates knowledge of the procedure allocation, the individual procedure assignment for each patient will be available. The codes will be available at the study center in sealed envelopes that are stored in a locked and secured area accessible only to those individuals authorized by the investigator. This procedure allows unblinding of individual subjects, without revealing codes of the entire study. The investigator will be able to determine which procedure a patient was given by opening the sealed envelope with the corresponding randomization number. The investigator must state the reason why the code was broken on the envelope and must date and sign it. At the end of the study, all sealed and unsealed envelopes must be accounted for.

### Outcome measures

**Fig. 1:**
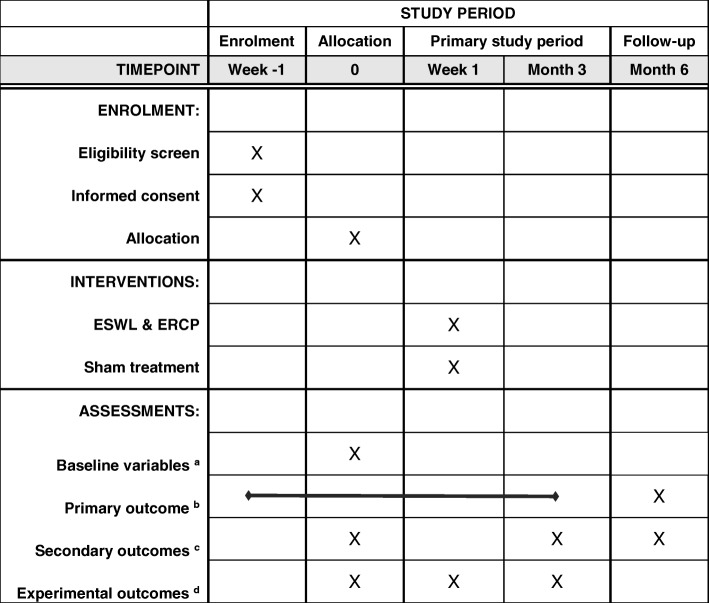
Schedule of enrollment, interventions, and assessments. ^a^Baseline variables: age; sex; etiology and duration of chronic pancreatitis (CP); history of acute pancreatitis; current alcohol and smoking status; complications of CP, including exocrine pancreatic insuficiency (EPI) and diabetes mellitus; cross-sectional imaging results; medications, including analgesics; and past history of endoscopic and/or surgical treatment. ^b^Primary outcome: Pain scores documented in a weekly pain diary based on a 0–10 visual analogue scale. ^c^Secondary outcomes: Pain scores after 6 months, > 30% pain relief, number of pain-free days, analgesic consumption, frequency of hospitalizations, quality of life (European Organization for Research and Treatment of Cancer Quality of Life questionnaire), pain and physical functioning composite scores (modified Brief Pain Inventory–short form), anxiety and depression scores, patient global impression of change, and complications. ^d^Quantitative sensory testing assessment parameters. *ERCP* Endoscopic retrograde cholangiopancreatography, *ESWL* Extracorporeal shock wave lithotripsy

At baseline, the following assessment parameters will be registered (Fig. 1): age and sex; etiology and duration of CP; past history of acute pancreatitis; current alcohol and smoking status; the presence of CP complications, including exocrine pancreatic insufficiency and diabetes mellitus; cross-sectional imaging results; current medications, including analgesics; and past history of endoscopic and surgical treatment. Data will be collected using a paper case report form and later entered into an electronic database with double data entry check.

The follow-up duration is 6 months from randomization with scheduled visits after 3 months (primary study period) and 6 months (follow-up). The questionnaires and pain diary used for collection of clinical endpoints are described in the previous sections on primary and secondary clinical endpoints. In addition, QST profiles will be recorded prior to intervention as well as 24–48 h after and 3 months after intervention. A schedule for the study assessment parameters is provided in Fig. 1.

### Quantitative sensory testing

#### Repetitive pinprick stimulation (temporal summation)

Recording of temporal summation to repetitive pinprick stimulations in the pancreatic and control areas (midline volar site of dominant forearm) will be employed using a 256-nm von Frey hair (Pin-Prick Stimulatoren; MRC Systems GmbH, Heidelberg, Germany). Pain ratings using a 0–10 numerical rating scale will be obtained after a single application and after the last application in a series of ten repetitive stimuli with an interstimulus interval of 1 s. For accurate timing of the stimuli, the procedure is guided by an auditory signal using a metronome. The difference between the last and the first pain ratings of the ten stimuli will be recorded as the temporal summation score.

#### Muscle pressure stimulation

The pressure pain detection threshold (PDT) and pain tolerance threshold (PTT) will be determined for the following skin dermatomes: C5 (clavicula), T10 ventral (upper epigastric area; pancreatic viscerotome), T10 back (pancreatic viscerotome), L1 (anterior superior iliac crest), and L4 (the quadriceps 15 cm above the patella). All lateralized pressure stimulations will be applied on the patient’s dominant side. An electronic pressure algometer (AlgoMed; Medoc Ltd., Ramat Yishai, Israel) with a probe surface area of 1 cm^2^ will be used for the pressure stimulations. Pressure will be increased in two separate sessions at a rate of 30 kPa/s until the PDT or PTT is reached. The assessment parameter is the pressure at the predefined sensory threshold measured in kilopascals.

#### Cold pressor test

The dominant hand is immersed in an ice-chilled water bucket (2.0 °C ± 0.3 °C). The patient will be told to remove the hand from the water after 2 min of immersion or sooner if the pain is intolerable. The patients rate the pain intensity for every 10 s during the cold pressor test using a 0–10 VAS. If the patient withdraws their hand sooner than 2 min because of intolerable pain, the VAS will be considered to be 10 for the remaining period of time, and the time for withdrawal of the hand will be noted.

#### Conditioned pain modulation

CPM is a clinically measurable form of descending pain modulation that can be induced experimentally by a conditioning stimulus (the cold pressor test) and quantified by applying a “test pain” (pressure stimulation on the nondominant quadriceps musculature 4 cm above the patella) before and after the conditioning stimulus [36]. The difference in pressure stimulus intensity (PTT) before and after the cold pressor test provides a quantitative index of the CPM capacity in the individual patient. The techniques used for pressure stimulation and cold pressor test described above will be combined to measure CPM.

### Pancreatic imaging

Cross-sectional imaging (CT and MRCP) will be used to evaluate the pancreatic morphology prior to study enrollment based on usual clinical practice. All patients enrolled in the study will have their imaging parameters reviewed and described by an expert radiologist (Dr. Ashirwad).

### Statistical analysis

The primary analysis of clinical endpoints will be by intention to treat, meaning that all randomized patients are included in their initially assigned study arm, regardless of adherence to the study protocol. Experimental endpoints will be evaluated by per-protocol analysis, meaning that only patients completing the experimental setup will be included. A repeated measures linear mixed effects model will be used for the primary analysis and will include terms for treatment group, assessment time point (week), and the interaction of treatment with assessment time point. Summary statistics of pain scores will be provided for the individual time points, and the difference in pain scores between groups after 3 months is considered the primary efficacy parameter. Subgroup and covariate analyses will be performed if applicable and in case differences in patient subgroups deemed clinically relevant are evident. Subsequent analyses directed at the secondary, experimental, and safety endpoints are analyzed using appropriate statistics, including mixed effect models, Fisher’s exact tests, and Student’s *t* tests or Wilcoxon rank-sum tests as appropriate. The predictive value of the QST profiles will be analyzed using logistic regression models.

We plan to conduct an interim analysis after approximately one-third of patients (*n* = 40) have been randomized and have completed 6 months of follow-up, using a two-sided significance test with the O’Brien–Fleming spending function and a type I error rate of 5%. An independent statistician, blinded to the treatment allocation, will perform the interim analysis. The statistician will report to an independent data and safety monitoring board (DSMB). The DSMB will have unblinded access to all data and will decide on the continuation of the trial and report to the internal review board of the Asian Institute of Gastroenterology. If there is a highly significant difference in improvement in pain in the treatment arm, then all subsequent patients will be subjected to this. The trial will not be stopped in case of futility, unless the DSMB advises otherwise during the course of safety monitoring.

### Monitoring and safety

Prior to trial initiation, a DSMB comprising a neurologist, an anesthesiologist, and a statistician (all from outside the study center) will be constituted. The DSMB will conduct periodic monitoring to ensure that the protocol and good clinical practice standards are followed. The monitors may review source documents to confirm that data recorded on case report forms are accurate. The investigator and institution will allow the DSMB and appropriate regulatory authorities direct access to source documents to perform this verification. The trial site may be subject to review by the institutional review boards, and/or to quality assurance audits performed by the DSMB, and/or to inspection by appropriate regulatory authorities. The DSMB will also conclude on the planned interim analysis.

There is no anticipated harm or compensation for trial participation. The ESWL and ERCP procedures on patients with CP are currently used in daily clinical practice, and participation in the study is not expected to be associated with an increased risk of adverse events compared with standard clinical care. Following ESWL, we have observed mild erythema at the site of stimulation. There has been a very infrequent incidence of acute pancreatitis after the procedure. Because ERCP is associated with an increased risk of post-procedure acute pancreatitis, means to decrease this risk (rectal indomethacin and preprocedural intravenous fluids) will be used in high-risk patients.

## Discussion

Pain is the primary symptom of CP and is associated with poor health-related outcomes, including reduced life quality, disability, and increased health resource use [37–39]. Pain treatment is unsatisfactory in a large proportion of patients and generally based on low-quality evidence in particular for invasive treatments (endoscopy and surgery) [14]. The SCHOKE (Extracorporeal Shock Wave Lithotripsy and Endotherapy for Pain in Chronic Pancreatitis) trial is a randomized, sham-controlled trial designed to determine if pancreatic duct decompression is an effective approach for obtaining pain relief in patients with CP. According to current guidelines, invasive procedures are recommended for pain treatment in the context of CP, and some practitioners even advocate for surgical treatment in the early phase of CP [32, 40, 41]. However, neither surgery nor endoscopic therapy has been thoroughly evaluated in sham-controlled trials [14]. The present study will, for the first time, provide sham-controlled evidence for the effectiveness of pancreatic duct decompression as a remedy to relieve pain in CP.

The mechanisms responsible for pain in patients with CP are multifactorial, and thus it is to be expected that no treatment can effectively relieve pain in all patients [42]. Identification of biomarkers that link pain mechanisms with specific treatment modalities is therefore an unmet need in the field, but there are currently few personalized approaches to pain treatment in CP [29]. For example, the presence, severity, and temporal nature of pain correlate poorly with imaging findings, and, as such, imaging can only be used to identify cases in which an invasive treatment is technically feasible, but it cannot be used to determine if patients will benefit from the planned treatment [7, 8, 43]. A QST protocol designed for characterization of pancreatic pain has previously shown effectiveness for the prediction of analgesic outcome in patients with CP and may serve as a useful biomarker for endoscopic treatment efficacy as well [24]. The SCHOKE trial therefore includes a QST testing paradigm specifically developed to assess and characterize pain in CP [29].

In conclusion, the SCHOKE trial is a randomized, sham-controlled trial that investigates whether pancreatic duct decompression is effective for obtaining pain relief in patients with painful CP.

## Trial status

Ethical approval has been obtained, and the trial was registered with ClinicalTrials.gov on May 25, 2019 (NCT03966781). The first patient was randomized by January 4, 2020, and recruitment is expected to be completed July 1, 2021.

## Supplementary information

**Additional file 1.** SPIRIT 2013 checklist: recommended items to address in a clinical trial protocol and related documents.

## Data Availability

Patients are coded using a numeric randomization code (anonymized), and only the principal investigators have access to this code. The source data are kept by the project leader for 15 years at the data center of the Asian Institute of Gastroenterology. All data generated or analyzed during this study will be included in the published results. Any data required to support the protocol can be supplied on request. The datasets analyzed during the current study are available from the corresponding author on reasonable request.

## Abbreviations

CP: Chronic pancreatitis
CPM: Conditioned pain modulation
CT: Computed tomography
DSMB: Data and safety monitoring board
ERCP: Endoscopic retrograde cholangiopancreatography
ESWL: Extracorporeal shock wave lithotripsy
MRCP: Magnetic resonance cholangiopancreatography
PDT: Pain detection threshold
PTT: Pain tolerance threshold
QST: Quantitative sensory testing
RCT: Randomized controlled trial
SCHOKE: Extracorporeal Shock Wave Lithotripsy and Endotherapy for Pain in Chronic Pancreatitis
SPIRIT: Standard Protocol Items: Recommendations for Interventional Trials
VAS: Visual analogue scale
